# Description of patients presenting with mental illness in emergency medical services: a retrospective observational study

**DOI:** 10.1186/s13049-025-01453-9

**Published:** 2025-08-13

**Authors:** Natalie Bergman, Aleksandra Jarling, Gabriella Boysen Norberg, Beatrice Alenljung, Magnus Hagiwara Andersson

**Affiliations:** 1 PreHospen: Centre for Prehospital Research, University of Borås, Allégatan 1, Borås, 50332 Sweden; 2 Faculty of Caring Science, Work Life and Social Welfare, University of Borås, Allégatan 1, Borås, 50332 Sweden; 3 School of Informatics, University of Skövde, Högskolevägen, Skövde, 54128 Sweden; 4 Fackulty of Health and Life Science, Univeristy of Linnaeus, Universitetsplatsen 1, Växjö, 352 52 Sweden

**Keywords:** Emergency medical service, Prehospital, Retrospective, Mental health problems, Mental illness

## Abstract

**Background:**

Mental illness is prevalent worldwide, creating a demand for Emergency Medical Service (EMS) assessments in mental illness, yet research on the epidemiology of patients with mental illness in the EMS is lacking in Sweden. This study aims to describe the patients presenting with symptoms of mental illness in the EMS and how they are assessed in the prehospital setting.

**Method:**

A retrospective observational study was conducted to identify patients assessed for symptoms of mental illness in the EMS in 2023. A total of 1,304 records met the inclusion criteria and were included in the study: [1] assessed in the EMS due to symptoms of mental illness and [2] over 13 years old. The data were analysed using IBM SPSS Statistic 28.

**Results:**

More females (54.3%) than men (45.7%) were assessed for mental illness (*p* = < 0.01). The median age was 39 years, with an interquartile range (IQR) of 32 years (*p* = < 0.01) and a total range of 13–91. Most patients were assessed once, with a range of 1 to 37 times. The initial priority of the patients was mainly Priority 1 (45.6%) or Priority 2 (49.9%). However, this shifted after the EMS assessment where most patients either recieved a lower priority or No priority [due to not being transported] (39.7%). The most common triage colour was Orange (21.4%), indicating the need for acute care, but four out of ten patients did not recieve a triage color (40.4%). The most frequent patients assessed by the EMS were suicide threats/attempts (45.2%) and intoxications (48.8%) with intoxication cases most likely to be hospitalised. The length of the stay in the hospital varied from 0 to 67 days but most patients were discharged within 24 h (6.8%) or admitted for 24 h (6.4%).

**Conclusion:**

Patients with mental illness are frequently assessed in the EMS, primarily for suicide threats/attempts and intoxication. However, few are admitted to the hospital, and many are not triaged, suggesting difficulties in referring patients with mental illness to the right level of care. The result may inform future studies assessing patients with mental illness in the EMS.

## Background

In this paper, *mental illness* is used to describe both psychiatric diagnoses and illnesses that cause stress and suffering, based on the WHO [1] definition of mental health. Mental illness is prevalent worldwide, creating a demand for the assessment of patients with mental illness in the Emergency Medical Service (EMS) [2, 3]. Today, one in eight people lives with a diagnosed mental disorder [4], and in the U.S, one in five people lives with mental illness [5]. Mental illness or disorder can present itself in various ways, including changes in behaviour, emotions, and thinking. It can lead to distress and difficulties in daily life, affecting family, work, and social activities, and cause significant suffering for the people experiencing it [6]. As a result of this prevalence of mental illness, patients presenting with symptoms of mental illness are now one of the top five reasons for ambulance dispatch [7]. These may include disorders and/or illnesses such as depression, anxiety, attempted suicide, psychosis, intoxication, and acute behavioural disturbance [2].

The EMS clinician’s role is to make quick assessments and provide treatment in the pre-hospital environment. They are responsible for patient care and ensuring the patient receives the right level of care by collaborating with other healthcare providers [8, 9]. Historically, the primary focus of EMS has been on emergencies and providing acute care. However, this focus has shifted, and EMS now provides care in both acute and non-acute situations, working in health promotion ways and minor injury care. Another significant change to the role of EMS is its expanding involvement in mental health care [10]. It is often the first healthcare provider that patients encounter when assessing the healthcare system and, thus plays a crucial role in the initial assessment of the patient’s mental illness [3, 10, 11].

People living with mental illness who need care should get help and access to the right care at the right time. Unfortunately, this is often not the case, and there is a worldwide gap between the demand for and availability of mental health services [12]. As a result, people with mental illness may call an ambulance to request help. Research indicates that people with mental illness are using the EMS with frequent calls and repeated use of an ambulance [7, 13, 14]. They are also more likely to return to the EMS within 72 h [7]. Patients who frequently use the EMS for mental illness and sometimes for non-acute problems are predominantly young adults and people who misuse alcohol [13, 15, 16]. Patients who misuse alcohol also tend to discharge themselves before receiving adequate care, leading to further contact with emergency care [15]. This takes time and resources away from emergencies and is sometimes seen by the public as a misuse of the EMS, which leads to patients with mental illness being sometimes seen as a burden to the system [3]. However, this issue reflects a larger systemic problem within healthcare, stemming from the disparity between the demand for mental health services and their availability, rather than a problem on the patient-individual level [12]. Long et al. [13] found that patients who frequently used the EMS knew that the EMS was not the right level of care for their illness and suffering, but they were unsure of where else to turn and wanted help quickly.

Despite the prevalence of patients with mental illness within EMS, descriptions of the patients are lacking. For example, there is a lack of data on who they are, what they suffer from, and how EMS clinicians assess them. A study conducted in Australia [14] found that 1 of 10 calls to the dispatch centre were due to mental illness, suggesting an underrepresentation of the actual numbers. Another study indicates that 40% of all calls to the dispatch centre are due to mental illness [17]. However, there are often discrepancies between the dispatch code, the paramedic’s initial assessment of the patient, and the outcome [2, 14]. This may relate to the dispatcher’s difficulty in determining the nature of the case over the phone because of the various ways mental illness can present itself [2, 6, 14]. Overall, research shows inconsistencies in the reported numbers of calls related to mental illness, ranging from 5% of calls to evidence of some component of mental illness in every call [3].

Patients with mental illness are known to require more time in emergency care than those with physical health issues [18], and such frequently use the EMS [7, 14]. However, research about the specific mental illness these patients seek help for, and the outcomes of the assessment is limited. This study is, to our knowledge, the first in Sweden to address this gap. EMS clinicians report an increasing demand from patients with mental illness [3, 10, 11, 18], but no overview of these patients has been conducted.

Given this background, the aim of this study was to describe the patients presenting with symptoms of mental illness in the EMS and how they are assessed in the prehospital setting.

## Methods

### Study design

This was a descriptive, retrospective observational study of patients assessed by the EMS for mental illness. Data were collected from EMS and hospital records. The study follows the Strengthening the Reporting of Observational Studies in Epidemiology (STROBE) guidelines [19].

### Settings and populations

This study was conducted in a western region in Sweden. The region serves 217, 000 inhabitants and had approximately 29 000 assignments during 2023.

In Sweden, the health care system is tax-funded and divided into 21 different regions responsible for health care within their areas. Each region retains self-governance regarding how they choose to structure and provide health care, except for matters specified by law, such as how the ambulance is staffed. By law, it is required that each ambulance is staffed with at least one registered nurse, often with a master’s degree in ambulance care/intensive care/anaesthesia care. The registered nurse is responsible for determining and deciding to which level of care the patient should be referred.

The most common triage systems used in both the EMS and emergency departments to determine the level of care in Sweden are Rapid Emergency Triage and Treatment System (RETTS) [20] and West coast System for Triage (WEST) [21]. In the region where the study was conducted, WEST is used by both the emergency department and the EMS. They are both similar with a five-level triage system (Red to Blue) and include emergency signs, symptoms, and vital signs as a guideline for triaging the patients. Combined with clinical judgment, current and medical history, the patient receives a triage colour [22]. *Red* indicates acute, life-threatening conditions; *Orange* acute, potentially life-threatening conditions; *Yellow* acute, not life-threatening conditions; *Green* less acute conditions and *Blue* not acute conditions. However, when it comes to symptoms of mental illness, except for intoxication, there are no symptoms or emergency signs; instead saying the EMS clinician has to use their clinical judgement when triaging [23]. This represents a significant disadvantage for ambulance clinicians, as symptoms and/or emergency signs play a crucial role in determining a patient’s triage colour. As a result, the use of WEST becomes challenging when assessing patients presenting with symptoms of mental illness. The advantage of using WEST is that research has shown that RETTS tends to triage the patients to a higher level, making it difficult to determine which require acute care. When 1500 patients were triaged according to both triage systems, it was found that WEST triaged patients to a lower level of care, and with no safety issues observed, supporting the use of WEST for more accurate determination of those requiring acute care [22]. In comparison to the triage approach, the emergency dispatch centre uses a different, numerical system to assess the patient’s condition, ranked from 1 to 4, where 1 means life-threatening, 2 potentially seriously ill, 3 the patient can wait, and 4 sickness transport, where no treatment is needed. The same system is used by the EMS clinicians when transporting the patient to the hospital [23]. The EMS clinicians also code the patient’s main reason for the EMS assessment.

When seeking care in Sweden and/or being admitted to a hospital, ICD-10 is the diagnostic system used. All patients, except stroke and hip fracture patients, who go directly to X-ray and ST elevation myocardial infarction patients who go directly to PCI, go through the emergency department before being admitted to a department.

### Data collection

**Fig. 1:**
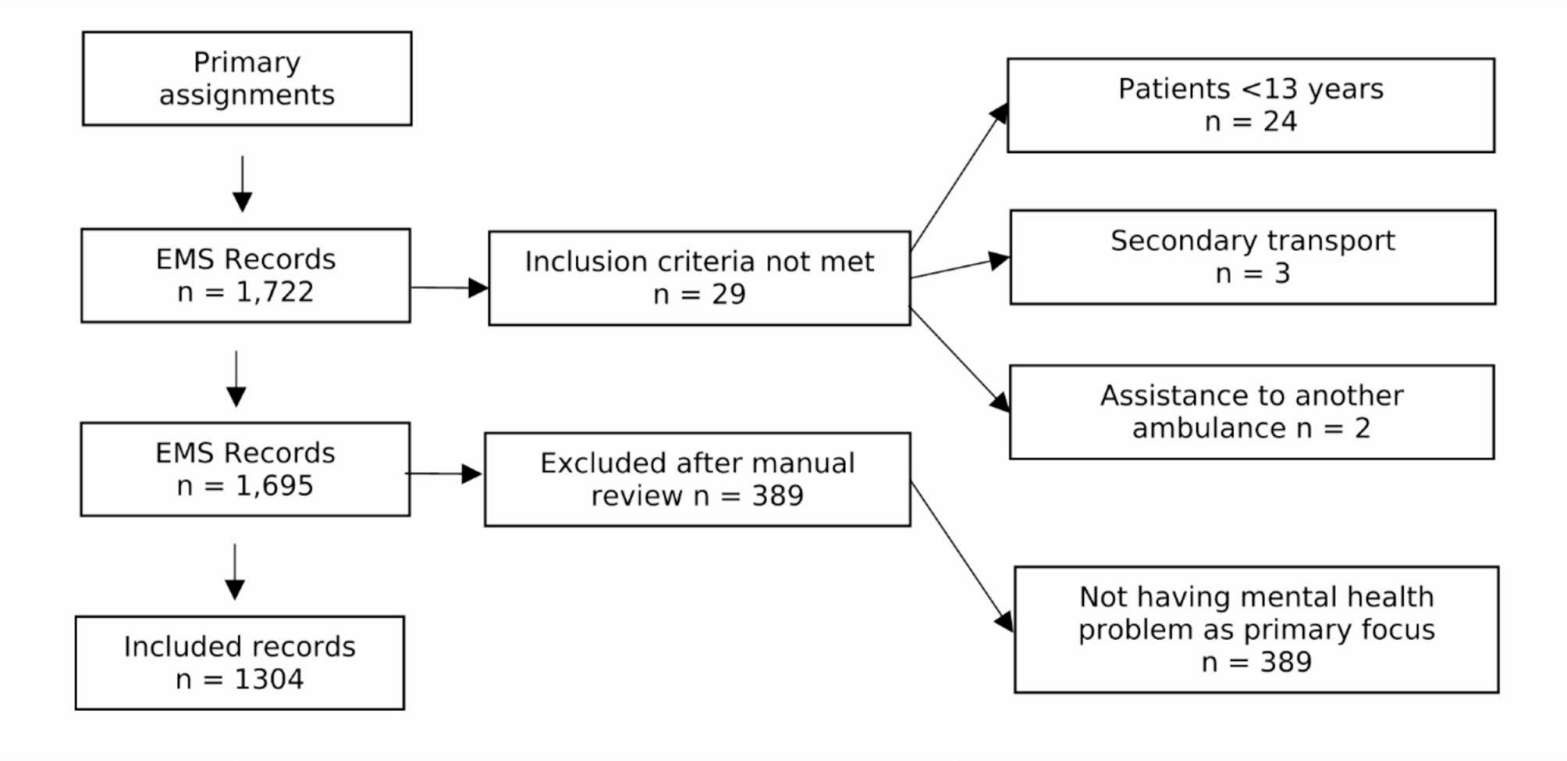
Flow chart of included patients

Data were collected in 2023 from EMS records (Ambulink) and hospital records (Melior) (Fig. 1). The data were initially searched in Ambulink using a variable list that included symptoms of mental illness, the assessment groups “psychiatry” and “poisoning,” and two free search terms: “suicide” and “psych.” The inclusion criteria for data extraction were patients assessed by the dispatch centre in the EMS service due to mental illness who were over 13 years old. The exclusion criteria were secondary transport, and assistance to another ambulance. In Melior, data were retrieved using a variable list that included hospitalisation [of the patients identified in the first search in Ambulink], duration of hospital length of stay, and whether patients sought care again within 72 h after discharge. When collecting data from Ambulink, the dispatch centres information is included with the EMS records as it provides the EMS clinicians with the first information the patient provides when they call 112. A total of 1,722 records were identified and constituted the dataset.

After receiving the dataset of 1,722 records, the first author manually reviewed it, and patients who were incorrectly included in the dataset based on the variable list were removed. This included 24 patients under 13 years, three of whom were secondary transport, and two who were assisted by another ambulance. Additionally, 389 records were excluded after manually reading the records for not having symptoms of mental illness as the primary focus. A patient could have several initial symptoms, e.g. suicidal threats and drug intoxication.

### Data analysis

Categorical variables are presented as counts and percentages, and continuous variables as median and range. Comparisons between groups were analysed using the Mann–Whitney U test for continuous variables and Fisher’s exact test for categorical variables. A *p*-value of < 0.05 was considered statistically significant for all tests. All analyses were conducted using SPSS 28.0 (SPSS Inc., Chicago, IL).

## Results

### Patient characteristics

In 2023, there were 1,304 assignments by the EMS service due to symptoms of mental illness (Table 1). This accounted for 4.5% of all assignments. There were more females (54.3%) than males (45.7%) assessed. The overall median age was 39 years. For females, the median age was 32 and for men, 44. When the dispatcher makes the initial assessment over the telephone regarding the patient’s reason for the EMS assessment, almost a third were coded due to suicidal threat (32.0%), and in 172 cases, suicide attempt (13.2%), as the initial reason. Of all patients, the initial assessment was that nearly a third were intoxicated with drugs or medication (30.0%) and almost one-fifth were intoxicated by alcohol (18.7%).

**Table 1:** Characteristics and frequency of patients assessed by the EMS

	*N* (%) 1304 (100)	Median	IQR	Min-Max	*P*-value
**Age**		39	32	13–91	< 0.01
Women		32	29	13–91	
Men		44	32	13–91	
**Gender**					
Women	708 (54.30)				
Men	596 (45.70)				
*Initial assessment by dispatcher					
Suicidal threats	417 (32.00)				
Drug or medication intoxication	392 (30.10)				
Alcohol intoxication	244 (18.70)				
Somatic symptoms caused by mental illness	189 (14.50)				
Anxiety or distress	188 (14.40)				
Suicide attempt	172 (13.20)				
Self harm	128 (9.80)				
Other, such as mania/psychosis	92 (7.10)				
Confusion	66 (5.10)				
Hallucinations	56 (4.30)				
Delusions	33 (2.50)				
**EMS assessments per patient**	1304	1	1	1–37	
**Multiple EMS assessment**					
No	820 (62.90)				
Yes	484 (37.10)				
**EMS reassessment < 72 h**					
No	1162 (89.10)				
Yes	142 (10.90)				
**Admitted to hospital**					
Yes	260 (19.90)				
No	1044 (80.10)				
**Somatic department**	210 (16.10)				
Medical emergency department	156 (12.00)				
Haematology and oncology department	10 (0.80)				
Children and Youth Department	10 (0.80)				
**Psychiatric department**	50 (3.80)				
Department 2, substance abuse	24 (3.80)				
Department 3, mood disorders	15 (1.30)				
Department 4, psychotic disorder	11 (0.80)				
**Top five diagnoses when patients admitted to hospital**	260 (19.90)				
T50.9 Other and unspecified drugs, medicaments, and biological substances	105 (8.10)				
F19.0 Mental and behavioural disorders due to multiple drug use and use of other psychoactive substances, acute intoxication	16 (1.20)				
F13.0 Mental and behavioural disorders due to use of sedatives or hypnotics, acute intoxication	12 (0.90)				
F10.0 Mental and behavioural disorders due to use of alcohol, acute intoxication	10 (0.80)				
F10.3 Mental and behavioural disorders due to use of alcohol, withdrawal state	7 (0.50)				
**Hospital length of stay**		3	2	0–67	
Less than a day	89 (6.80)				
One day	83 (6.40)				
Two days	25 (1.90)				
Three days	14 (1.10)				
Four days	15 (1.20)				
< Four days	34 (2.50)				
**New hospital contact < 72 h**					
Yes	92 (7.10)				
No	168 (12.90)				
Psychiatric emergency department	49 (3.80)				
Psychiatric departments	16 (1.30)				
Somatic departments	27 (2.00)				

* One patient could have several initial assessed symptoms of mental illness

Most patients were assessed by the EMS once during 2023, but there was a range from one assessment per patient up to 37 times. In 484 cases (37.1%), the patient had multiple assessments, and in 142 cases (10.9%), the patient called for an ambulance again within 72 h hours of the previous assessment.

Of the patients assessed by the EMS due to symptoms of mental illness, one in five (19.9%) was admitted to the hospital. In most cases, they were admitted to a somatic department (16.1%), and in three out of four cases to the medical emergency department. When the patients were admitted to the psychiatric department (3.8%), they were admitted directly to the specific department that most aligned with the patient’s mental illness, where the substance abuse department was the most common (1.8%). The largest group by far comprised patients under the influence of other and unspecified drugs, medicines, and biological substances (8.1%). The other four most common ICD codes also involved any type of intoxication or withdrawal symptoms.

Length of stay in the hospital varied from 0 to 67 days, but most patients were discharged within 24 h (6.8%) or admitted for 24 h (6.4%). There were 92 patients who sought care again at the hospital within 72 h (7.1%) of discharge, whereas most patients went to the psychiatric emergency department (3.80%).

### EMS assessment and management

When the dispatcher prioritises patients with mental illness, almost all patients are labelled as priority 1 (45.6%) or a priority 2 (49.9%) (Table 2). Time spent on scene by the EMS has a median of 32 min, and the total prehospital time has a median of 80 min. During patient assessments, EMS clinicians contacted the psychiatric emergency department for counselling in approximately one out of every five cases (21.0%).

**Table 2:** Dispatch priority, EMS assessment, and management

	*N* (%)	Median	Min-Max
**Dispatch priority**	1304 (100)		
Priority 1	594 (45.60)		
Priority 2	651 (49.90)		
Priority 3	54 (4.10)		
Priority 4	5 (0.40)		
**Time at scene (minutes)**	1294*	32	0–180
**Total prehospital time (minutes)**	1303**	80	5–249
**Prehospital counselling**			
Psychiatric emergency department	274 (21.00)		
**Triage colour**			
Colour missing	526 (40.30)		
Red	111 (8.50)		
Orange	279 (21.40)		
Yellow	168 (12.90)		
Green	207 (15.90)		
Blue	9 (0.70)		
Dead on scene	4 (0.30)		
**EMS priority level**			
Priority 1	133 (10.20)		
Priority 2	462 (35.40)		
Priority 3	165 (12.70)		
Priority 4	26 (2.00)		
No priority/non-transported	518 (39.70)		
**Level of care referral**			
Somatic emergency department	554 (42.50)		
Psychiatric emergency department	282 (21.60)		
Other***	88 (6.60)		
Self-care advice	155 (11.90)		
Care was recommended but declined	111 (8.50)		
Self-care advice and primary care	40 (3.10)		
Municipal intervention	42 (3.20)		
Outpatient psychiatric clinic	16 (1.20)		
**Assessment code by EMS**	1304 (100)		
Intoxication	369 (28.30)		
Psychiatry	660 (51.30)		
Medicine	29 (2.20)		
Other	64 (4.90)		
Circulation	28 (2.10)		
Neurology	17 (1.3)		
Orthopaedics	7 (0.50)		
Infection	6 (0.50)		
Surgery	66 (5.10)		
Injuries	16 (1.20)		
Gynaecology	1 (0.10)		
General Symptoms	15 (1.20)		
Respiration	4 (0.30)		
Injuries and accidents	1 (0.10)		
Assessment Group Missing	12 (0.90)		
****ICD 10 code after EMS assessment			
F99 Mental disorder, not otherwise specified	669 (51.30)		
T50.9 [Poisoning with] Other and unspecified drugs, medicaments, and biological substances	181 (13.90)		
T65.9 Toxic effect of unspecified substance [alcohol & drugs]	113 (8.70)		
T40.6 [Poisoning with] Other and unspecified narcotics	38 (2.90)		

* 10 records miss time stamps

** 1 record missed time stamp

*** Other referrals such as social services, police, patient absconds/stays at home, MET (medical emergency [home] team), deceased

**** Only the four largest groups are explained in the table

After the EMS assessment, the predominant group of patients recieved no priority (39.7%) since they were not referred to the hospital. Of those who were referred, priority 2 (35.4%) was the most common priority level, followed by priority 3 (12.7%) and priority 1 (10.2%). When triaging the patients, more than a third of the patients were not assigned a triage colour (40.3%). The most common colour was Orange (21.4%) followed by Green (15.8%) and then Yellow (12.9%). Most patients were referred to the Somatic emergency department (42.4%) and the Psychiatric emergency department (21.6%), while one in ten patients was referred to self-care (11.9%); a small group of patients were recommended care but declined (8.5%).

The main reason for more than half of the patients’ EMS assessments was psychiatric symptoms (51.3%) and almost a third were assessed due to intoxication (28.3%). Looking at ICD10, over half were coded in as mental disorders (51.3%), with other three largest groups involving intoxication of various kinds.

## Discussion

The main findings of this study show that intoxication and suicide threats/attempts are the most common symptoms of patients in the EMS with mental illness. Moreover, one-fifth of those assessed by EMS clinicians were hospitalised. Overall, this seems to be a difficult patient group to triage under the current triage system since four out of ten patients were not assigned a triage colour.

Previous research conducted in Asia [16] and Scotland [15] has shown that patients who are intoxicated with alcohol and/or drugs are common in the EMS. This was likewise evident in the present study. Holzer and Minder [24] gathered data over ten years, looking at patients who had intoxication as the primary reason for assessment in the EMS, and they found an increase over time by approximately 5% per year. In the present study, patients who were intoxicated stood for the highest number of hospitalisations, although the number is low in relation to the summation of patients assessed due to intoxication. Smith-Bernardin, Kennel and Yeh [25] conducted a study on patients who were intoxicated by alcohol and examined whether the emergency department is the right referral for these patients. They found the sobering centre as a safe alternative to the patients being referred to emergency departments. In Sweden though, sobering centres exist in only six regions but is something being discussed at the government level due to the increasing number of intoxications [26]. In an interview study, patients who use the EMS when intoxicated expressed guilt over using ambulance resources but at the same time, most patients reported an unwillingness to change their alcohol misuse and depend on external support, leading to the continued frequent use of the EMS service [16]. These contradictions of feeling guilty but not accepting care could be explained by the shame and feeling of being judged for alcohol misuse that patients express [27]. One might assume that the suffering these patients experience is due to this conflict of interest. The question remains, however, whether the emergency department is the right level of care for these patients, as being transported to and being in, the emergency department could cause additional stress for the patient. On the other hand, you could assume that it at the same time brings relief as the patients get access to care quickly, which could increase the patient’s sense of safety.

Another finding that stood out in the present study was suicide threats/attempts, with suicide threats being the most common initial assessment by dispatchers. Worldwide, more than 720,000 people die every year from suicide, and for every suicide, there are many previous threats and attempts [28]. In Sweden, 1617 people were either confirmed to have committed suicide (82%) or suspected of doing so (18%) in 2023 [29]. It is therefore unsurprising that EMS clinicians meet these patients often, something supported by previous research [30, 31]. In many cases, the suicide threats/attempts are in combination with intoxication in intentional ways to try to commit suicide [30, 31]. However, in the present study, not one patient got suicide as an ICD 10 code assigned after EMS assessment, although we can conclude that these patients got “F99 Mental Disorder” as the diagnosis code, as the number is consistent with the number of patients assessed initially due to suicide threat/attempt. These patients did not tend to be admitted to the hospital, based on the findings that none of the patients got suicide ideation or suicide attempt as an ICD 10 code when admitted, which is concerning since, as stated earlier, for every suicide, there are several threats and attempts [28]. Research [32] has also shown that the feeling of hopelessness may be a contributing factor to repeated suicide attempts, which can be interpreted as a desire to be seen and get help, rather than a desire to die. One might assume the suffering these patients experience when calling for help and not receiving care. Patients who called for the EMS while being in a suicidal process describe in an interview study the suffering they are in, in such a vulnerable place, feeling suicidal, and at the same time they struggle with the stigma and fear of being judged [33]. Drawing the conclusion they were coded with “F99 Mental Disorder” and were referred to the emergency department but then not admitted makes you wonder where these patients should receive care, for their suicidal ideation, that is sustainable in the long run, to avoid trips to the emergency departments.

In line with this, neither intoxication nor suicide threats/attempts seems to be sufficient to allow admission to the hospital, where the present study shows that only one-fifth of the patients referred to the emergency department were admitted to the hospital. However, it is important to clarify that in the present study, we do not know if the patients wanted to be hospitalised. For example, patients who were in a suicidal process described that the most important thing for them was to be seen, heard, and acknowledged as human beings in their suffering and receive validation of their feelings [33]. It still raises the question of whether the emergency department was the right place for referral or where these patients should be referred to have their care needs met. In the present study, approximately 30% of the patients got either Red or Orange as a triage colour, indicating the need for acute care. The other patients got a lower triage colour, indicating there was no need for acute care. Previous research shows that many patients with mental health problems have sought care in primary care but did not receive help for their illness, leading the patients to call for an ambulance [13, 14]. Patients with mental illness describe how important it is to be acknowledged as a person and receive care for their mental illness. When being dismissed, this could increase the suffering [18]. One might assume the helplessness and suffering these patients feel, not receiving the care they need or being admitted anywhere [hospital, primary care]. However, in the present study, the EMS clinicians only referred the patients to primary care in 3% of the cases as most patients were transported to the emergency department. This could be because of a lack of competence and confidence in the EMS clinicians when assessing patients with mental health problems [10, 11], leading the EMS clinicians to play it safe and take the patient to the emergency room for instant care, instead of referring to primary care [34]. It could also be due to the lack of decision support and guidelines regarding where to refer the patient, leaving it up to the EMS clinician to go with their gut feeling and intuition [11]. These factors in combination could be the cause of why patients are referred to the emergency department when there is not always a need for acute care [35]. Another suggestion for why EMS clinicians refer patients to the emergency department when acute care is not always needed is the desire to help these patients as EMS clinicians witness their difficulty with mental health problems and receiving help from primary care and therefore drive the patient to the emergency department in the hope of helping them get the ball rolling [11]. On the other hand, another reason why patients are not referred to the emergency department, as outlined in the previous study, could be because of the difficulty in triaging patients with mental health problems as the current triage system WEST [21] is mainly focused on physical health problems, not mental health problems. The fact that there are no indications for the different triage colours within mental health problems; rather, it states that there are no individual warning signs for mental illness and that the EMS clinicians need to make their own assessment based on the clinical assessment, with special consideration for suicidal thoughts, psychotic symptoms, and aggressivity. This could be why 40% of the patients in the previous study were missing a triage colour or, referred to the emergency department, and then were not admitted; in other words, there is a lack of decision support and guidelines making it hard to assess the patient’s to the right level of care.

Patients with mental illness are already struggling with stigmatisation in society and describe how they felt ashamed when calling for an ambulance due to mental health problems as they were afraid of not being taken seriously [27]. At the same time, the present study, strengthened by several other studies [13, 14, 17, 35] suggests that the EMS and emergency department are not the right place for these patients. The question remaining is how these patients should receive care and where the EMS clinicians should refer them to alleviate their suffering. This needs to be addressed in future research.

### Method discussion

When extracting the data from EMS records, one inclusion criterion was that the patients had to be over 13 years old. This age was determined by the fact that, in Sweden, health services consider patients at the age of 13 to be cognitively developed enough to start taking responsibility for care contacts; thus parents have limited access to their medical records. We believe this cognitive development also reflects on their actions and feelings when it comes to mental illness, making over 13 years an age at which we could identify as many patients as possible in as wide an age range as possible.

During the manual review, 389 patients were excluded for not having mental health problems as the primary focus. These patients were included in the original dataset because they met the inclusion criteria. However, upon manual review, it was found that a patient could have, for example, called an ambulance for a fracture and had a mental illness in their medical history. However, as mental illness had nothing to do with the assessment, they were excluded.

### Strengths and limitations

The main strength of this study was a large database of 1300 patients assessed specifically for mental illness. It provides a diversity to the data which increases generalisability and reliability. However, a limitation was that mental illness can disguise itself as physical illness. This means there may be an underreporting in the actual statistics of those assessed by the EMS for mental illness, as data were retrieved from specific variables that were directly related to mental illness.

## Conclusion

This study indicates that every twentieth patient an EMS clinician encounters and assesses today is due to symptoms of mental illness. Most patients were assessed once but in four out of ten cases, the patients had previously been assessed for mental illness. Suicide threats/attempts and intoxications were the most frequent causes for patients to visit the EMS. However, few patients assessed for suicide threats/attempts are hospitalised while the most common reason for hospitalisation was the patients intoxication. EMS clinicians are responsible for treating patients and referring them to the right level of care, and to help them, they rely on a triage system. Unfortunately, this system does not include psychiatric symptoms as it is mainly focused on somatic symptoms and diseases. This was also evident in the study, where four out of ten patients were not triaged using the current triage system. This result may be useful for future studies addressing the assessment of patients with mental illness problems in the EMS.

## Data Availability

No datasets were generated or analysed during the current study.

## Abbreviations

EMS: Emergency Medical Services. Refers to the ambulance service and clinicians
RETTS: Rapid Emergency Triage and Treatment System
WEST: West coast System for Triage
